# Serious Adverse Events in Adult ICU Patients: A Substudy of the PATIENCE Cohort

**DOI:** 10.1111/aas.70382

**Published:** 2026-10-30

**Authors:** Vera Crone, Morten Hylander Møller, Anders Granholm, Anders Perner, Waleed Alhazzani, Abdulrahman Al‐Fares, Johanna Hästbacka, Marlies Ostermann, Carmen A. Pfortmueller, Ricard Ferrer, Annika Reintam Blaser, Eric Keus, Wojciech Szczeklik, Mette Krag

**Affiliations:** ^1^ Department of Intensive Care Holbæk Hospital Holbæk Denmark; ^2^ Department of Intensive Care Copenhagen University Hospital–Rigshospitalet Copenhagen Denmark; ^3^ Department of Clinical Medicine University of Copenhagen Copenhagen Denmark; ^4^ Section of Biostatistics, Department of Public Health University of Copenhagen Copenhagen Denmark; ^5^ Research and Innovation Institute Ministry of Defense Health Services Riyadh Saudi Arabia; ^6^ Critical Care and Internal Medicine Department, College of Medicine Imam Abdulrahman Bin Faisal University Riyadh Saudi Arabia; ^7^ Department of Anaesthesia, Critical Care Medicine and Pain Medicine, Al‐Amiri Hospital Ministry of Health Kuwait City Kuwait; ^8^ Department of Intensive Care Tampere University Hospital, Wellbeing Services County of Pirkanmaa and Tampere University Tampere Finland; ^9^ Department of Critical Care King's College London, Guy's and St. Thomas' Hospital London UK; ^10^ Department of Intensive Care Inselspital, Bern University Hospital and University of Bern Bern Switzerland; ^11^ Department of Intensive Care Vall d'Hebron Hospital Universitari, Vall d'Hebron Barcelona Hospital Campus Barcelona Spain; ^12^ Department of Anaesthesiology and Intensive Care University of Tartu Tartu Estonia; ^13^ Department of Intensive Care Medicine Lucerne Cantonal Hospital Lucerne Switzerland; ^14^ Department of Critical Care University Medical Center Groningen Groningen the Netherlands; ^15^ Center for Intensive Care and Perioperative Medicine Jagiellonian University Medical College Krakow Poland; ^16^ Department of Anaesthesiology, Surgery and Trauma Centre Copenhagen University Hospital – Rigshospitalet Copenhagen Denmark

## Abstract

**Introduction:**

Prokinetic agents are used to enhance upper gastrointestinal motility, but adverse events related to these agents are poorly described. We aimed to provide detailed epidemiological data on predefined serious adverse events (SAEs) in a cohort of adult intensive care unit (ICU) patients with and without prokinetic treatment. We hypothesised that all predefined SAEs would occur in both patients treated and those not treated with prokinetic agents, and that a higher number of SAEs would occur among patients treated with prokinetic agents for more than three consecutive days.

**Methods:**

This substudy of the inception cohort study “Prokinetic agents in adult intensive care unit patients” included acutely admitted adult ICU patients. The prokinetic agents assessed (exposures) were erythromycin, metoclopramide, domperidone, and prucalopride. The primary outcome was the proportion of patients experiencing at least one episode of five pre‐specified SAEs during ICU stay (cardiac arrhythmias, cardiac arrest, vomiting with aspiration, diarrhoea, extrapyramidal symptoms). Secondary outcomes were (1) proportion of patients experiencing each SAE, (2) the proportion of patients experiencing any of the SAEs after prokinetic treatment stratified by longest consecutive treatment duration, (3) time from index ICU admission to first SAE and (4) time from prokinetic initiation to first SAE. All analyses were descriptive, summarising SAE proportions and timing overall and according to prokinetic exposure.

**Results:**

Among 1440 adult ICU patients, 710 SAEs occurred in 354 patients (24.6%), most commonly cardiac arrhythmias (199/1440, 13.8%). Across the exposure groups, the proportion of patients experiencing one or more SAEs ranged from 23.0% to 42.5%. Among patients treated with prokinetic agents for > 3 consecutive days, 14/59 (23.7%) experienced a SAE, which was similar to the proportion observed among patients not receiving prokinetic agents (291/1.253, 23.2%). Most SAEs occurred on median Day 2 after ICU admission and on median Day 3 after prokinetic initiation.

**Conclusion:**

One in four adult ICU patients experienced at least one SAE, with cardiac arrhythmias being the most frequent. Among patients receiving prokinetic agents, the proportion experiencing one or more SAEs ranged from 23.0% to 42.5% across treatment groups. The findings from this study may help inform the design of a future clinical trial.

**Editorial Comment:**

This analysis presents reported serious adverse event observed in an ICU trial cohort assessing gastrointestinal prokinetic drug effects. Serious adverse events were reported frequently in both the drug treatment groups and the comparison (no prokinetic drug) group. Types of serious adverse events reported are presented.

## 1 Background

Delayed gastric emptying is a common challenge in intensive care unit (ICU) patients and may reduce the uptake of nutrition [1, 2]. Prokinetic agents are used to enhance upper gastrointestinal motility, and the most commonly used are metoclopramide, a dopamine receptor antagonist, and erythromycin, a motilin receptor agonist [3].

The adverse effects of prokinetic agents in the ICU are poorly described, but safety concerns are well established in other settings [3]. Both metoclopramide and erythromycin may prolong the QT interval and increase the risk of cardiac arrhythmias [3]. Erythromycin additionally inhibits CYP3A4, which further increases the risk of arrhythmias when combined with other drugs [3, 4]. Erythromycin has also been associated with reductions in systolic blood pressure in healthy subjects, and its antimicrobial properties raise concerns regarding antibiotic resistance and antibiotic‐associated diarrhoea [5, 6, 7].

Metoclopramide may cause neurological adverse effects, including extrapyramidal symptoms and tremor. However, the risk is poorly quantified, and neurological adverse events related to prokinetic use in the ICU have not been systematically reported [1].

The overlap between potential drug‐related events and symptoms of underlying illness further complicates the assessment of adverse events in critically ill patients [8, 9, 10].

We recently conducted the international ‘Prokinetic agents in adult intensive care unit patients’ (PATIENCE) cohort study to describe the use of prokinetic agents in adult ICU patients and evaluate the association between prokinetic use and the occurrence of serious adverse events (SAEs) [11]. The purpose of this descriptive substudy was to provide a detailed overview of the predefined SAEs in the entire cohort, including the occurrence and timing relative to ICU admission and initiation of prokinetic treatment. We hypothesised that all predefined SAEs would occur in both patients exposed and unexposed to prokinetic agents, and that a higher frequency of SAEs would be observed among patients receiving prokinetic treatment for more than three consecutive days.

## 2 Methods

### 2.1 Design

This substudy of the PATIENCE cohort was conducted according to a pre‐published protocol [12]. The manuscript has been prepared in accordance with the Strengthening the Reporting of Observational studies in Epidemiology (STROBE) statement (checklist available in Table S1) [13]. Approval for this substudy was obtained from the Danish Data Management Region of Zealand (EMN‐2024‐03449).

### 2.2 The PATIENCE Study

The PATIENCE study was an international inception cohort study designed to describe the use of prokinetic agents and to explore potential associations between prokinetic use and patient‐important outcomes, including days alive out of the ICU and hospital within 90 days, SAEs, and mortality. The study included 1440 patients from 56 ICUs across 11 countries [11]. Each site selected a 14‐day inception period between August 2024 and March 2025, during which all acutely admitted adult ICU patients were enrolled and followed daily throughout their ICU stay for up to 90 days [14].

### 2.3 Population

All acutely admitted adult (≥ 18 years) ICU patients were eligible for inclusion. Patients with a planned ICU admission, those who declined informed consent when required, or those already included in the database were excluded. All patients in the primary PATIENCE cohort were eligible for this substudy.

### 2.4 Data Collection and Key Definitions

Data were collected as part of the primary PATIENCE cohort and are described in detail elsewhere [11, 14]. For this substudy, we used baseline variables and daily ICU data from the primary cohort to evaluate the use of prokinetic agents and selected predefined SAEs. Data on predefined SAEs were collected prospectively from patients' medical records on a daily basis throughout the ICU stay. The PATIENCE study recorded predefined SAEs predefined in the protocol [14]: cardiac arrest, cardiac arrhythmias, extrapyramidal symptoms, vomiting with clinically significant pulmonary aspiration, and diarrhoea exceeding 1000 mL/day. Complete definitions are available in Supporting Information S2 and S3. These predefined events are hereafter referred to as SAEs. Prokinetic treatment was defined as treatment with erythromycin, metoclopramide, domperidone, or prucalopride, regardless of dose or duration. Because only two patients were treated with domperidone and none received prucalopride, this substudy focuses on metoclopramide and erythromycin. Patients were assigned retrospectively to one of four mutually exclusive exposure groups based on the timing of prokinetic treatment relative to SAE occurrence. Patients who never received a prokinetic agent or who experienced all SAEs before prokinetic initiation were assigned to the no‐prokinetic group. All remaining patients were assigned according to the first prokinetic agent received (erythromycin, metoclopramide, or combination treatment). Accordingly, patients who experienced a SAE on or after treatment initiation were assigned to the corresponding treatment group even if they had experienced any SAE before initiation. Treatment group assignment remained unchanged even if treatment was subsequently changed.

### 2.5 Outcomes

The primary outcome for this substudy was the proportion of patients experiencing one or more predefined SAEs during ICU stay stratified according to the 4 predefined exposure groups (Table 1).

Secondary outcomes included: (1) the proportion of patients experiencing each of the 5 predefined SAEs during ICU stay stratified according to the 4 predefined exposure groups; (2) the proportion of patients with any SAE after receiving prokinetic agents stratified in two groups by longest consecutive treatment duration (≤ 3 vs. > 3 ICU days); (3) time from index ICU admission to first SAE (regardless of prokinetic use); and (3) time from prokinetic initiation to first SAE during index admission (Table 1).

**TABLE 1:** Overview of exposure groups.

Groups
SAE^a^ without or before prokinetic treatment
2 SAE after/at metoclopramide initiation
3 SAE after/at erythromycin initiation
4 SAE after/at combination (erythromycin + metoclopramide) initiation
Patients receiving treatment in ≤ 3 consecutive days
Patients receiving treatment in > 3 consecutive days
All patients experiencing at least one SAE during index ICU stay, regardless of prokinetic treatment
All patients receiving a prokinetic agent during index ICU stay and experiencing their first SAE after treatment initiation

*Note:* Patients treated with prokinetic agents were grouped according to their first prokinetic treatment: Erythromycin, metoclopramide, or a combination of the two. Subsequent treatment did not alter the initial group assignment.

Abbreviations: ICU, intensive care unit; SAE, serious adverse events.

^a^ SAEs: Cardiac arrest, cardiac arrhythmias, extrapyramidal symptoms, vomiting with clinically important aspiration, and diarrhoea exceeding 1000 mL/day.

The three‐day cutoff was chosen based on guidelines from *the European Society for Clinical Nutrition and Metabolism*, which recommend limiting prokinetic treatment to 72 h [15].

### 2.6 Statistics

#### 2.6.1 Sample Size

The sample size was fixed by the primary cohort, which included 1440 patients.

Preliminary data from the PATIENCE cohort [11] indicated that approximately 25% of patients experienced at least one SAE, and 14% experienced a SAE on or after the first day of prokinetic treatment [12].

#### 2.6.2 Analyses

Baseline characteristics are described for all patients and secondarily stratified by the occurrence of one or more SAEs. All data are presented descriptively, with categorical data presented as counts and percentages, and continuous variables presented as medians with interquartile ranges (IQR).

All analyses were performed using R version 4.4.1 (R Core Team, R Foundation for Statistical Computing, Vienna, Austria).

#### 2.6.3 Missing Data

Missing data are reported as counts and percentages. For patients transferred to non‐participating ICUs, no events were assumed to have occurred during the time spent in the non‐participating ICUs.

## 3 Results

### 3.1 Baseline Characteristics and Primary Outcome

In the included cohort (*n* = 1440), 56.9% were male, the median age was 64 years (IQR 49–75), and 47.6% were admitted from emergency departments. Circulatory failure was the most frequent reason for ICU admission (26.1%). Overall, 187 (13%) received prokinetic agents during their ICU stay (Table 2). Of these, 125 were prescribed metoclopramide, 40 had erythromycin, and 20 received a combination of both agents. Baseline characteristics of patients with and without a SAE are presented in Supporting Information S4 Table S1.

**TABLE 2:** Baseline characteristics of the overall cohort.

	Overall *N* = 1440
Age (years)	64 (49–75)
Male sex	819 (56.9%)
Comorbidities	
Pulmonary disease	275 (19%)
IHD or HF	267 (19.1%)
Chronic renal failure	135 (9.4%)
Chronic liver failure	68 (4.7%)
Diabetes (type 1 or 2)	389 (27.1%)
Haematologic malignancy or metastatic cancer	127 (8.8%)
Admitted from	
ED	686 (47.6%)
General ward	403 (28.0%)
OR	269 (18.7%)
ICU	82 (5.7%)
Surgery during hospital admission	
Elective surgery	174 (12.1%)
Emergency surgery	356 (24.8%)
Abdominal surgery	188 (13.1%)
Surgery before hospital admission	
Bariatric surgery	14 (1.0%)
Illness severity	
SMS‐ICU^a^	17 (13–22)
SMS‐ICU predicted 90%‐day mortality (%)^b^	25.3 (16.5–40.1)
Main reason for ICU admission	
Neurological condition	286 (19.9%)
Respiratory failure	359 (24.8%)
Circulatory failure	376 (26.1%)
Renal failure	51 (3.5%)
Liver failure	23 (1.6%)
GI bleeding	38 (2.6%)
Other	307 (21.3%)
Treatments prior to ICU admission	
Prokinetic agents^c^	62 (4.3%)
Parenteral nutrition^d^	62 (4.3%)
Country	
Denmark	641 (45%)
Saudi Arabia	193 (13%)
Sweden	114 (7.9%)
Finland	95 (6.6%)
Poland	90 (6.3%)
United Kingdom	75 (5.2%)
Spain	70 (4.9%)
Estonia	63 (4.4%)
Netherlands	55 (3.8%)
Kuwait	30 (2.1%)
Switzerland	14 (1.0%)

*Note:* Missing baseline data (7 patients, 0.5% in total): blood pressure: two patients (0.1%); information on elective surgery: one patient (0.1%); information on one comorbidity: Three patients (0.2%); information on parenteral nutrition: one patient (0.1%). Continuous variables are presented as medians with interquartile range (IQR) and categorical variables as counts and percentages.

Abbreviations: ED, emergency department; GI, gastrointestinal; HF, heart failure; ICU, intensive care unit; IHD, ischaemic heart disease; OR, operating or recovery room.

^a^ Median raw scores (IQR).

^b^ Predicted 90‐day mortality based on Simplified Mortality Score for the Intensive Care Unit (SMS‐ICU) (an illness severity score ranging from 0 to 42).

^c^ Treatment with erythromycin, metoclopramide, domperidone or prucalopride.

^d^ Treatment with parenteral nutrition at the time of ICU admission.

The cohort included 1440 patients of whom 354 (24.6%) experienced 710 SAEs during ICU stay. Most SAEs occurred in patients not exposed to prokinetic agents: 291 patients (82.2% of those with SAEs) either never received a prokinetic agent (277 patients) or experienced all SAEs prior to treatment initiation (14 patients). The remaining 63 patients who experienced SAEs (17.8%) experienced one or more SAEs on prokinetic initiation or thereafter. Of these, 15 patients had also experienced a SAE before treatment initiation. In 11 of these patients the same type of SAE occurred both before and after treatment initiation: cardiac arrhythmias (*n* = 6), vomiting with aspiration (*n* = 2), diarrhoea (*n* = 2) and cardiac arrest (*n* = 1).

In total, 503/710 (70.8%) SAEs occurred in patients who never received prokinetic agents or had all SAEs before treatment initiation. An additional 53/710 (7.5%) SAEs occurred before prokinetic initiation among the 15 patients who also experienced a SAE on or after treatment initiation. These patients were assigned retrospectively to the corresponding prokinetic exposure group.

The remaining 154/710 (21.7%) SAEs occurred on or after prokinetic initiation. Among these 154 post‐initiation events, 98 (63.6%) occurred after metoclopramide initiation, 35 (22.7%) after erythromycin initiation, and 20 (13.0%) after initiation of combination therapy. Two patients received domperidone as the first agent; one had no SAEs, and one had vomiting with clinically significant aspiration on the day of treatment initiation.

Figure 1 shows the proportion of patients experiencing at least one SAE across the four groups. Cardiac arrhythmia was the most commonly reported predefined SAE (199/1440 patients, 13.8%); 24 (12.1%) of those experiencing cardiac arrhythmia had received a prokinetic agent before the event. Cardiac arrest was the second most frequent SAE, occurring in 158/1440 (11.0%) patients; 22 (14.0%) of those experiencing cardiac arrest had received prokinetic treatment before the event (Supporting Information S5, Table S2).

**FIGURE 1:**
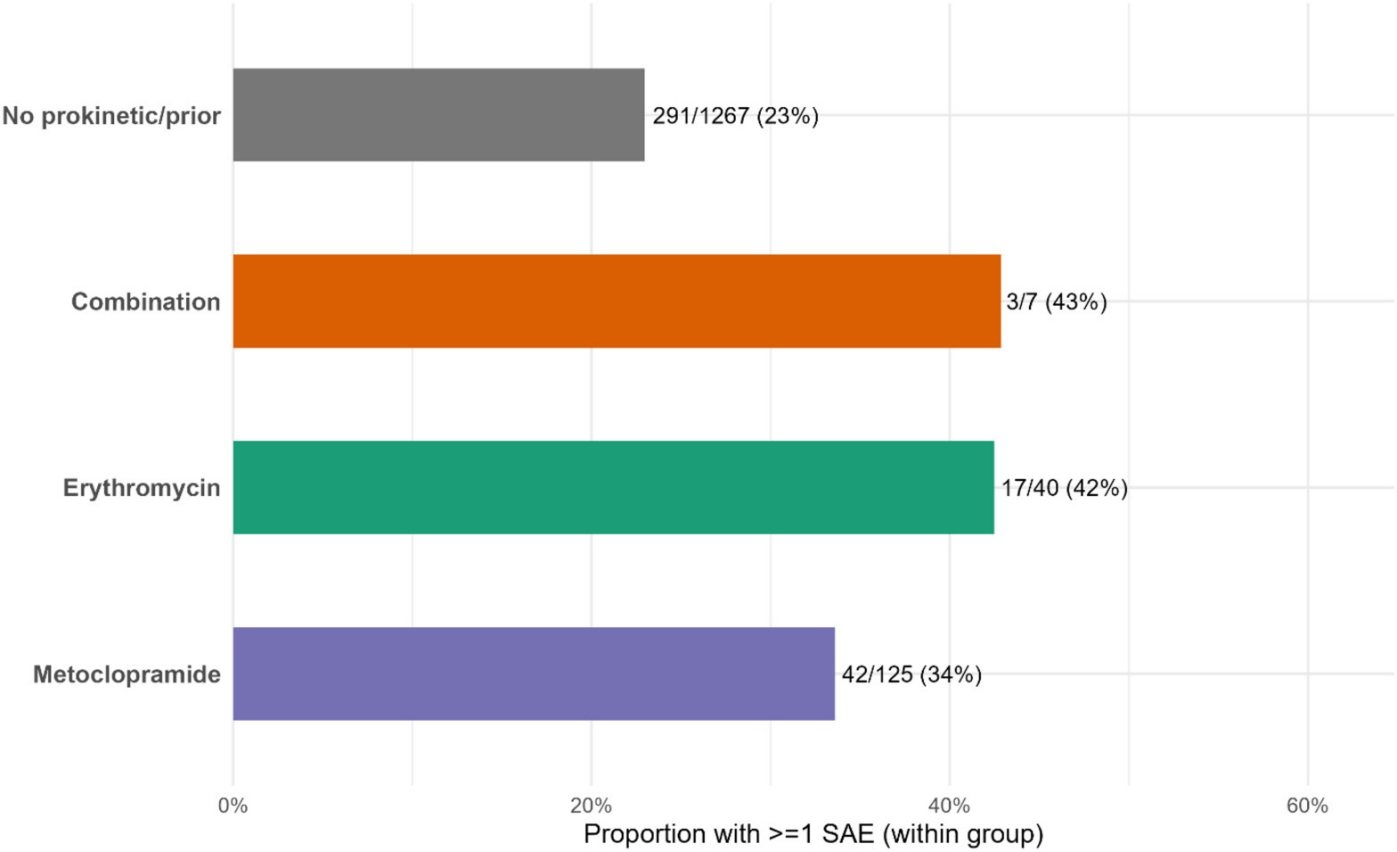
Proportion of patients with one or more SAEs. Proportion of patients with ≥ 1 serious adverse event (SAE) by first prokinetic group: Metoclopramide; erythromycin; combination (both agents on the first treatment day); and a fourth group comprising patients who never received metoclopramide/erythromycin or whose SAEs all occurred before the first dose. Denominators are all patients in each group; for treated groups, SAEs are counted if occurring on or after the first dose (including same day). Two patients received domperidone and are excluded from the figure. Fifteen patients experienced SAEs both before and on or after prokinetic initiation and were assigned to the corresponding treatment group.

### 3.2 Secondary Outcomes

Across most SAEs, the highest patient‐level proportions were observed among patients who had erythromycin as their first prokinetic agent, except for vomiting with aspiration, which occurred only in patients who received metoclopramide as their first prokinetic agent or in those not receiving prokinetic agents (Figure 2). Cardiac arrhythmias and cardiac arrest occurred at similar proportions in metoclopramide‐treated patients and in those not receiving prokinetic agents. In contrast, diarrhoea was more frequent after treatment with prokinetic agents (10%–14%) than among patients not treated with prokinetic agents (2.9%). Extrapyramidal symptoms were rare overall, reported in 11 patients (0.8%).

**FIGURE 2:**
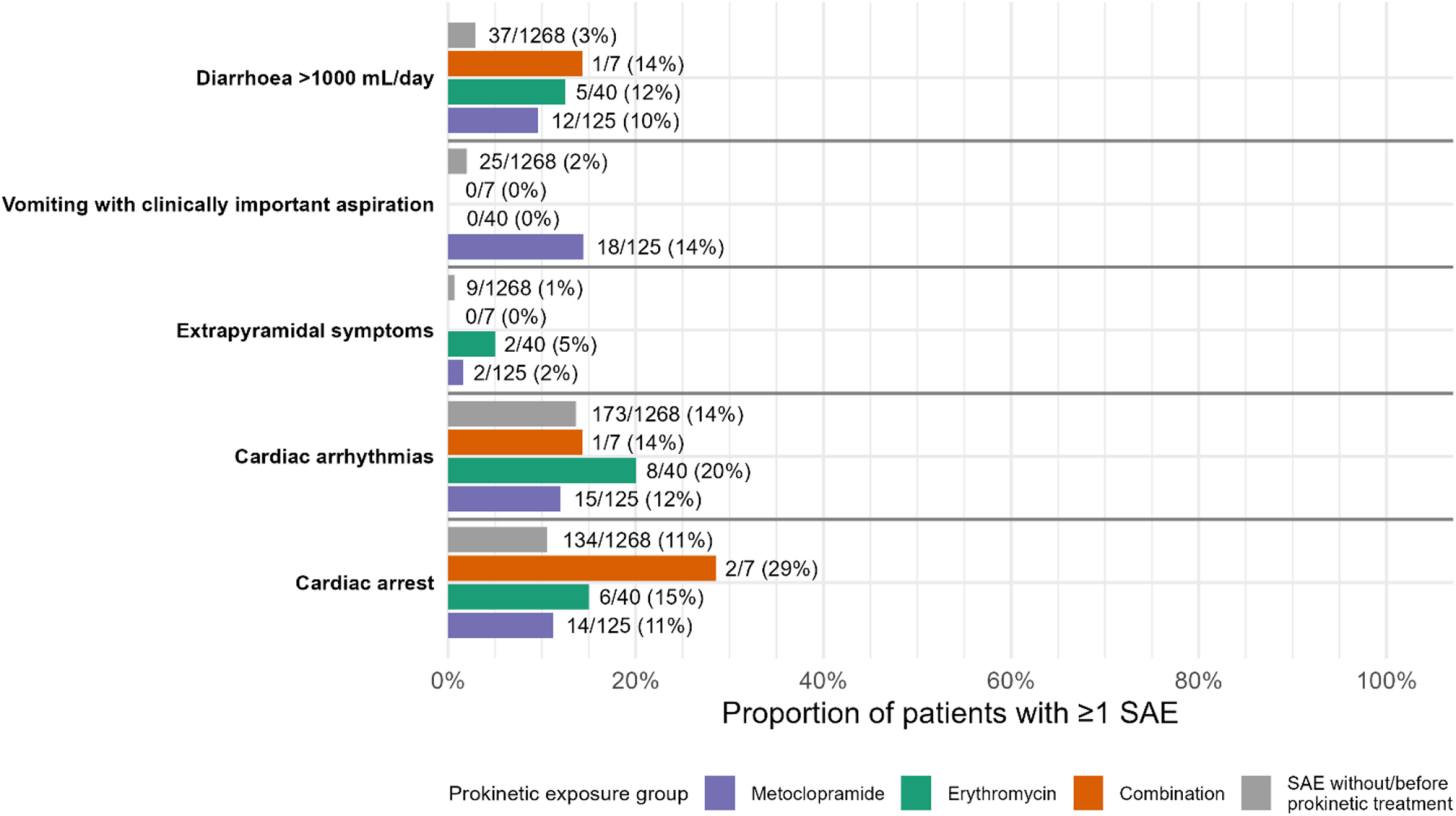
Proportion of patients experiencing each of the predefined SAEs within the 4 exposure groups. Proportion of patients with each of the predefined serious adverse event (SAE) by exposure group. For patients receiving prokinetic agents, SAEs are counted from the first prokinetic dose onward. The no‐prokinetic group includes patients who were never treated with prokinetics or had all SAEs before initiation of prokinetic treatment. Patients may appear in multiple SAE types but are counted only once per type. Percentages are calculated within exposure groups. Fifteen patients experienced SAEs both before and on or after prokinetic initiation and were assigned to the corresponding treatment group; 11 experienced the same SAE type in both periods: Arrhythmias (*n* = 6), vomiting with aspiration (*n* = 2), diarrhoea (*n* = 2) and cardiac arrest (*n* = 1).

### 3.3 SAEs and Treatment Duration

A total of 59 patients were treated for more than three consecutive days at some point during admission, most commonly with metoclopramide alone (24/59, 40.7%) or in combination with erythromycin (22/59, 37.3%). In this group, 14 out of 59 (23.6%) experienced one or more SAEs after 3 days of treatment. Among those treated for ≤ 3 consecutive days, 100 out of 128 (78.1%) were treated with metoclopramide and 23 out of 128 (18.0%) with erythromycin. Of these 128 patients, 28 (21.9%) experienced at least one SAE after treatment cessation. The proportion of patients experiencing cardiac arrest, cardiac arrhythmias, and diarrhoea was higher in the group treated for > 3 consecutive days (Supporting Information S6, Table S3).

### 3.4 Time From ICU Admission to First SAE

Among the 354 patients who experienced at least one SAE, 340 (96.0%) had their first SAE during the index ICU stay, whereas 14/354 (4.0%) had their first SAE during a readmission to the ICU. Overall, most SAEs during the index stay occurred early after ICU admission: 136 out of 340 (40.0%) on day one and 68 out of 340 (20.0%) on day two, independent of prokinetic treatment (Figure 3). The first SAE occurred on median day 2 (IQR 1–4) following ICU admission.

**FIGURE 3:**
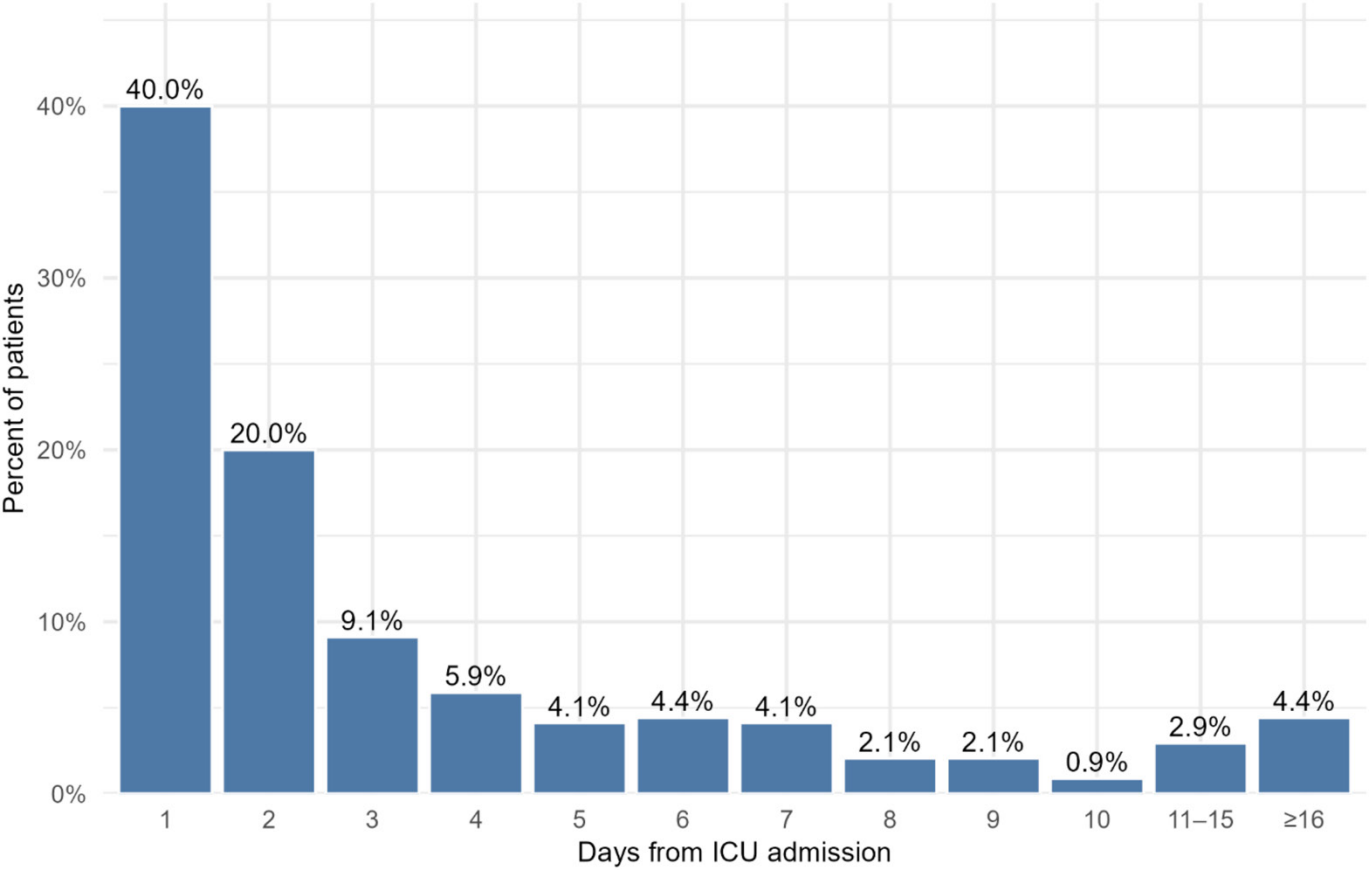
Time from index intensive care unit admission to first serious adverse event (days). Time from index intensive care unit (ICU) admission to first predefined serious adverse event (SAE). Bars show the percentage of patients with a first SAE on each ICU day. The denominator is all patients who experienced at least one of the 4 predefined SAE during the index admission (*N* = 340) regardless of prokinetic use. Each patient contributes once at their first SAE.

### 3.5 Time From Onset of Prokinetic Treatment to First SAE

Among patients who received prokinetic agents, 31.9% experienced their first SAE on the day treatment was initiated, and 14.9% on day four after initiation (Figure 4). Among patients whose first SAE occurred after prokinetic initiation, the median day of first SAE was 3 (IQR 1–6) after initiation.

**FIGURE 4:**
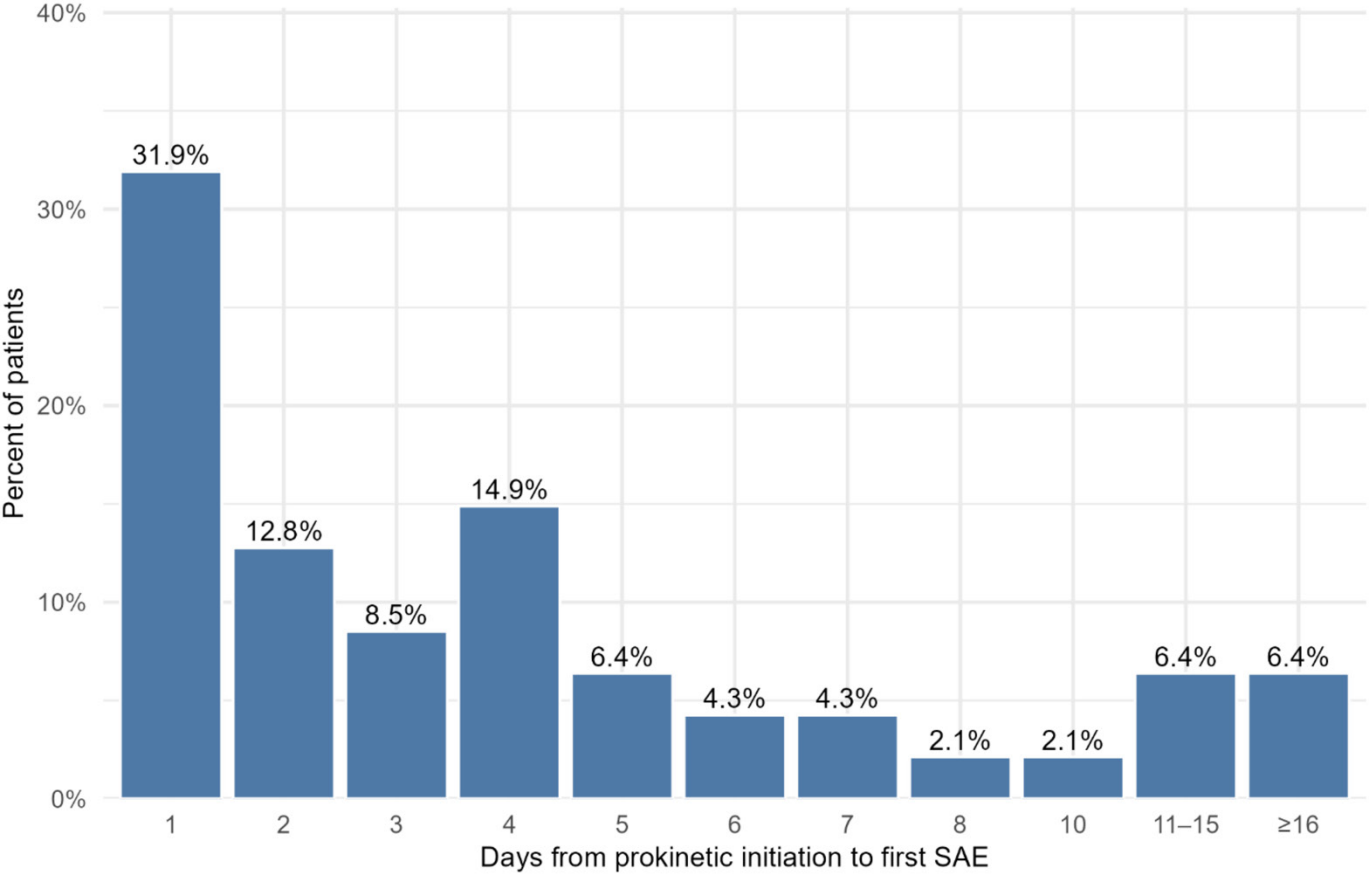
Time from initiation of prokinetic treatment to first serious adverse event (days). Time from prokinetic initiation during the index intensive care unit (ICU) admission to first predefined serious adverse event (SAE) among patients whose first SAE occurred on the day of or after treatment initiation (*n* = 48). Bars show the percentage of patients experiencing their first SAE on each day after initiation (Day 1 = day of initiation). Each patient is counted once. Prokinetic initiation is defined as the first ICU day with any metoclopramide, erythromycin, or domperidone (including combination therapy).

### 3.6 Missing Data

Overall, in the primary cohort < 5% patients had missing data for one or more variables [11]. Seven patients (0.5%) had missing data for one or more baseline variables. Two patients (0.1%) had a single ICU day with missing SAE data (Supporting Information S7, Tables S4 and S5). Sixty‐six patients (4.6%) were transferred to a non‐participating ICU and, hence, were assumed not to have had any SAEs for the remaining ICU time.

## 4 Discussion

In this substudy of the PATIENCE cohort of 1440 adult ICU patients [11], 24.6% experienced one or more of the predefined SAEs during ICU stay regardless of prokinetic treatment. The proportion of patients experiencing one or more SAEs was highest in those treated with erythromycin, and this pattern was consistent across most SAEs, except for vomiting with aspiration, which was observed only in patients not receiving prokinetic agents or those who received metoclopramide. Among patients treated with prokinetic agents, the proportion experiencing one or more SAEs was higher among those treated for more than 3 consecutive days. Most SAEs occurred in the first few days after ICU admission and shortly after prokinetic initiation.

Cardiac adverse events were more frequently reported in patients treated with erythromycin, consistent with its known potential to prolong the QT interval [16]. This risk may be amplified in the ICU, where co‐administration of other QT‐prolonging drugs or CYP3A4 inhibitors such as ketoconazole and amiodarone is common [16, 17]. In a prospective observational study of 51 ICU patients receiving 200 mg of erythromycin twice a day, treatment was found to statistically significantly prolong the QTc interval at 15 min and at 24 h. However, no QT‐related arrhythmias were seen [4].

In our study, gastrointestinal adverse events were more commonly reported among those receiving prokinetic agents. The proportion of patients with diarrhoea was highest in the group receiving combination therapy, although numbers were small, increasing uncertainty. This finding aligns with a prospective observational study of 180 ICU patients treated with prokinetics for feeding intolerance, who reported diarrhoea in 40% after treatment initiation, with higher rates on combination therapy (49%) than with metoclopramide (32%) or erythromycin alone (30%) [6]. However, diarrhoea in the ICU is multifactorial, with causes such as underlying illness, enteral nutrition, or concomitant antibiotics [9], making attribution to a specific drug difficult and leaving reverse causation and confounding by indication plausible.

Vomiting with clinically significant aspiration was proportionally higher among patients receiving metoclopramide, likely reflecting confounding by indication as metoclopramide is often initiated to treat nausea and vomiting [18].

Extrapyramidal symptoms were rare across all groups, which may reflect short‐term use and difficulties in recognising these symptoms in critically ill patients [1].

Overall, the proportion of patients experiencing one or more SAEs was slightly higher among those treated for more than three consecutive days. The current *European Society for Clinical Nutrition and Metabolism* guidelines recommend limiting prokinetic treatment to 72 h due to concerns about tachyphylaxis [6, 18]. However, this finding should be interpreted with caution, as patients receiving prolonged treatment are also likely to have longer ICU stays and consequently a longer time at risk for experiencing a SAE. Our findings nevertheless underscore the importance of evaluating the safety of prokinetic use, including extended use in adult ICU patients, in future randomised clinical trials.

There is a considerable risk of confounding by indication and reverse causation in this study, as patients with greater baseline illness severity and more comorbidities were both more likely to receive prokinetic agents and more prone to SAEs, independent of any treatment effect. This likely explains the short interval from ICU admission and from prokinetic initiation to SAE occurrence.

In addition, treatment decisions reflect contraindications, clinicians' risk assessments, and variable thresholds for initiating treatment, which may further introduce differences between treated and untreated patients. This may be particularly relevant for erythromycin, as its selection as first‐line treatment may reflect underlying factors associated with the occurrence of SAEs.

A serious adverse reaction (SAR) is an SAE assessed to be related to the investigational medicine [19]. In this study, we reported SAEs which, unlike SARs, do not rely on any assessment of causality, and no causal inferences were made. The occurrence of SAEs both before and after treatment initiation in some patients further illustrates the challenges of distinguishing events arising from underlying critical illness from potential SARs [19, 20]. These data highlight the need for randomised clinical trials to enable causal inferences and guide their design by identifying patient‐important outcomes, including relevant SAEs.

The strengths of this study reflect those of the PATIENCE study, including a large international ICU cohort, prospective data collection, and data completeness. Although this analysis is exploratory, it was conducted according to a predefined protocol published before data analysis from the primary study, thereby increasing transparency [12].

The study also comes with limitations. First, we reported on SAEs in the context of an observational study, and no conclusions can be drawn about a causal relationship between prokinetic agents and SAEs. Second, the timing analyses were descriptive and did not account for treatment cessation. Third, despite the overall large cohort, the number of patients receiving prokinetic agents and experiencing SAEs was relatively small, limiting the precision of subgroup analyses. Fourth, we registered data only on days spent in participating ICUs, and potential events occurring outside these units were not included. Fifth, data collection focused on broad categories; for instance, cardiac arrhythmias were recorded as any new arrhythmia, without information on type or duration, and no data were available on the suspected aetiology of the SAEs. Furthermore, we did not collect data on concomitant medications that may have influenced the occurrence of specific SAEs, such as laxatives, which could have contributed to the development of diarrhoea. Finally, although definitions of SAEs were prespecified in the protocol and implemented in the database, some degree of subjectivity cannot be excluded.

## 5 Conclusion

In this substudy of 1440 ICU patients, 25% experienced one or more of the predefined SAEs during their ICU stay, regardless of prokinetic treatment, with cardiac arrhythmias and cardiac arrest being the most common.

Among patients treated with prokinetic agents, the proportion experiencing one or more SAEs was highest in those treated with erythromycin, and among those treated for more than three consecutive days. Data from this substudy may help inform the design of future clinical trials evaluating the safety and efficacy of prokinetic agents in ICU patients.

## Author Contributions

**Vera Crone:** conceptualisation, methodology, formal analysis, validation, investigation, visualisation, writing first draft, data curation and project administration. **Morten Hylander Møller:** conceptualisation, methodology, and supervision. **Anders Granholm:** conceptualisation, methodology, formal analysis, and supervision. **Anders Perner:** conceptualisation and methodology. **Waleed Alhazzani:** conceptualisation and methodology. **Mette Krag:** conceptualisation, methodology, writing first draft and supervision. All authors: Investigation, critical review, and editing and approval of manuscript.

## Funding

The authors have nothing to report.

## Conflicts of Interest

The Department of Intensive Care at Rigshospitalet—Copenhagen University Hospital (M.H.M., A.G., A.P.) has received funding from the Independent Research Fund Denmark, Novo Nordisk Foundation, and Sygeforsikringen “danmark” outside the submitted work. A.R.B. is holding a grant from the Estonian Research Council (PRG3121). Ricard Ferrer has received honoraria from Shionogi M.S.D. Gilead Menarini Thermofisher Viatris A.O.P. outside the submitted work.

## Supporting information


**Table S1:** Baseline characteristics. Missing baseline data (7 patients, 0.5% in total): blood pressure: two patients (0.1%); information on elective surgery: one patient (0.1%); information on one comorbidity: three patients (0.2%); information on parenteral nutrition: one patient (0.1%) (detailed in supplementary material 7).
**Table S2:** SAEs in the cohort. Overview of SAEs in the cohort regardless of prokinetic treatment. In the number of events, the same patient may appear multiple times if they have experienced more than one SAE.
**Table S3:** Number of patients experiencing each predefined SAE after prokinetic treatment, stratified by treatment duration. Numbers represent the number of patients with each SAE, stratified by treatment duration. Percentages are calculated relative to the total number of patients in each duration group (≤ 3 days: *n* = 138; > 3 days: *n* = 49). A patient may contribute to multiple SAE types but is counted only once per type. For the ≤ 3‐day group, SAEs were counted only if they occurred after treatment discontinuation; for the > 3‐day group, SAEs were counted from treatment Day 4 onward.
**Table S4:** Missing baseline variables. Seven patients had one missing baseline variable (0.5%). For those with missing blood pressure, no measurement was recorded within the first 24 h of ICU admission. Only variables with missing values are included in the table.
**Table S5:** Missing daily variables. Three patients (0.2%) had one or more daily variables missing. In one patient, all life support variables were missing for a single intensive care unit day. Only variables with missing values are shown002E.

## Data Availability

Anonymised data supporting the findings of this study can be made available from the corresponding author (VC) upon reasonable request, subject to applicable permissions and data sharing agreements.
