# Supplementary material for: Serious Adverse Events in Adult ICU Patients: A Substudy of the PATIENCE Cohort
Source: Acta Anaesthesiol Scand. 2026 Oct 30;71(1):e70382. doi: 10.1111/aas.70382 (PMC13722535; doi:10.1111/aas.70382)
Supplement: Supplementary file 1 [file AAS-71-0-s001.docx]

**Supplementary material**

**Supplement to: Serious adverse events in adult ICU patients: A sub-study of the PATIENCE cohort**

**Authors:** Vera Crone, Morten Hylander Møller, Anders Granholm, Anders Perner, Waleed Alhazzani, Abdulrahman Al-Fares, Johanna Hästbacka, Marlies Ostermann, Carmen A Pfortmueller, Ricard Ferrer, Annika Reintam Blaser, Eric Keus, Wojciech Szczeklik, Mette Krag

***Corresponding Author:***

*Vera Crone, MD, Department of Intensive Care, Holbæk Hospital, Smedelundsgade 60, 4300 Holbæk, Denmark*

*Email : veracrone@gmail.com*

Table of contents

[S1 STROBE checklist	3](#_Toc237936139)

[S2 Definitions	6](#_Toc237936140)

[S3 Simplified Mortality Score for the Intensive Care Unit (SMS-ICU)	10](#_Toc237936141)

[S4 Baseline table	11](#_Toc237936142)

[S5 Overview of SAEs	12](#_Toc237936143)

[S6 SAEs and treatment duration	12](#_Toc237936144)

[S7 Missing data	13](#_Toc237936145)

[References	14](#_Toc237936146)

### S1 STROBE checklist

STROBE Statement—checklist of items that should be included in reports of cohort studies

|  | Item No. | Recommendation | Page  No. |  |
| --- | --- | --- | --- | --- |
| **Title and abstract** | 1 | (*a*) Indicate the study’s design with a commonly used term in the title or the abstract | 1 |  |
|  |  | (*b*) Provide in the abstract an informative and balanced summary of what was done and what was found | 3 |  |
| Introduction | | | |  |
| Background/rationale | 2 | Explain the scientific background and rationale for the investigation being reported | 4 |  |
| Objectives | 3 | State specific objectives, including any prespecified hypotheses | 4 |  |
| Methods | | | |  |
| Study design | 4 | Present key elements of study design early in the paper | 5-7 |  |
| Setting | 5 | Describe the setting, locations, and relevant dates, including periods of recruitment, exposure, follow-up, and data collection | 5 |  |
| Participants | 6 | (*a*) Give the eligibility criteria, and the sources and methods of selection of participants. Describe methods of follow-up | 5 |  |
|  |  | (*b*) For matched studies, give matching criteria and number of exposed and unexposed | NA |  |
| Variables | 7 | Clearly define all outcomes, exposures, predictors, potential confounders, and effect modifiers. Give diagnostic criteria, if applicable | 5-6 + Supplementary material 2 |  |
| Data sources/ measurement | 8* | For each variable of interest, give sources of data and details of methods of assessment (measurement).  Describe comparability of assessment methods if there is more than one group | 5-6  NA |  |
| Bias | 9 | Describe any efforts to address potential sources of bias | N/A |  |
| Study size | 10 | Explain how the study size was arrived at | 6 |  |

Continued on next page

| Quantitative variables | 11 | Explain how quantitative variables were handled in the analyses. If applicable, describe which groupings were chosen and why | 6-7 |  |
| --- | --- | --- | --- | --- |
| Statistical methods | 12 | (*a*) Describe all statistical methods, including those used to control for confounding | 6-7 |  |
|  |  | (*b*) Describe any methods used to examine subgroups and interactions | N/A |  |
|  |  | (*c*) Explain how missing data were addressed | 7 |  |
|  |  | (*d*) If applicable, explain how loss to follow-up was addressed | 7 |  |
|  |  | (*e*) Describe any sensitivity analyses | N/A |  |
| Results | | | |  |
| Participants | 13* | (a) Report numbers of individuals at each stage of study—eg numbers potentially eligible, examined for eligibility, confirmed eligible, included in the study, completing follow-up, and analysed | 6 |  |
|  |  | (b) Give reasons for non-participation at each stage | N/A |  |
|  |  | (c) Consider use of a flow diagram | - |  |
| Descriptive data | 14* | (a) Give characteristics of study participants (eg demographic, clinical, social) and information on exposures and potential confounders | 8 + table 2 |  |
|  |  | (b) Indicate number of participants with missing data for each variable of interest | 10 + Supplementary material 7 |  |
|  |  | (c) Summarise follow-up time (eg, average and total amount) | - |  |
| Outcome data | 15* | *Cohort study*—Report numbers of outcome events or summary measures over time | 8-9 |  |
| Main results | 16 | (*a*) Give unadjusted estimates and, if applicable, confounder-adjusted estimates and their precision (eg, 95% confidence interval). Make clear which confounders were adjusted for and why they were included | 9 (descriptive data) |  |
|  |  | (*b*) Report category boundaries when continuous variables were categorized | - |  |
|  |  | (*c*) If relevant, consider translating estimates of relative risk into absolute risk for a meaningful time period | - |  |

Continued on next page

| Other analyses | 17 | Report other analyses done—eg analyses of subgroups and interactions, and sensitivity analyses | 8-9 |  |
| --- | --- | --- | --- | --- |
| Discussion | | | | |
| Key results | 18 | Summarise key results with reference to study objectives | 10 |  |
| Limitations | 19 | Discuss limitations of the study, taking into account sources of potential bias or imprecision. Discuss both direction and magnitude of any potential bias | 11-13 |  |
| Interpretation | 20 | Give a cautious overall interpretation of results considering objectives, limitations, multiplicity of analyses, results from similar studies, and other relevant evidence | 12-13 |  |
| Generalisability | 21 | Discuss the generalisability (external validity) of the study results | 12 |  |
| Other information | |  | | |
| Funding | 22 | Give the source of funding and the role of the funders for the present study and, if applicable, for the original study on which the present article is based | 1 |  |

*Give information separately for cases and controls in case-control studies and, if applicable, for exposed and unexposed groups in cohort and cross-sectional studies.

**Note:** An Explanation and Elaboration article discusses each checklist item and gives methodological background and published examples of transparent reporting. The STROBE checklist is best used in conjunction with this article (freely available on the Web sites of PLoS Medicine at http://www.plosmedicine.org/, Annals of Internal Medicine at http://www.annals.org/, and Epidemiology at http://www.epidem.com/). Information on the STROBE Initiative is available at [www.strobe-statement.org](http://www.strobe-statement.org).

NA: not applicable.

### S2 Definitions

Baseline patient and admission characteristics

**General patient data**

- Sex (male/female)
- Age (years)
- Hospital admission date (dd.mm.yyyy)
- ICU admission date and time (dd.mm.yyyy and hh:mm)

**Source of admission**

- Emergency department or prehospital setting (Any Accident/Emergency /Casualty/Acute department in same or another hospital or direct admission to the ICU by an ambulance service or similar)
- General ward (Any location in the same or other hospital not covered in the other 3 categories)
- Operating or recovery room (Any surgical theatre, endoscopy and angiography suite and any recovery facilities observing patients following invasive procedures)
- Another ICU

**Main reason for ICU admission**

- Neurological condition
- Respiratory failure
- Circulatory failure
- Renal failure
- Liver failure
- GI bleeding
- Other

**Surgery before ICU admission**

- **Elective surgery**

*Definition: Surgery during current hospitalisation scheduled 24 hours or more before surgery*

- **Emergency surgery**

*Definition: Surgery during current hospitalisation scheduled 24 hours or less (including surgery performed at other hospitals) before surgery*

- **Abdominal surgery**

*Definition: Any abdominal surgery (Any laparotomy or laparoscopic surgery involving the organs or structures within the abdominal cavity including structures in the pelvic region) during current hospital admission prior to ICU admission. (Endoscopic procedures are NOT considered surgery in this setting).*

- **History of bariatric surgery**

*Definition: Gastric bypass, gastric sleeve, gastric banding, gastric balloon*

**Comorbidities**

1. **Chronic pulmonary disease**

*Definition: Any history of chronic obstructive pulmonary disease, asthma, or other chronic lung disease or if the patient was treated at time of hospital admission with any relevant drug indicating chronic pulmonary disease, e.g. albuterol, levalbuterol, salmeterol, formoterol, arformoterol, indacaterol, vilanterol, olodaterol, tiotropium, aclidinium, umeclidinium, glycopyrronium, budesonide and fluticasone.*

2. **History of severe heart failure (NYHA 3-4) or myocardial infarction**

*Definition: History of previous myocardial infarction, invasive intervention for coronary artery disease, stable or unstable angina, NYHA class 3 or 4 or measured LVEF < 40%.*

*NYHA class 3: marked limitation of physical activity. Comfortable at rest. Less than ordinary activity causes fatigue, palpitation, shortness of breath or chest pain.*

*NYHA class 4: symptoms of heart failure at rest. Any physical activity causes further discomfort.*

3. **History of chronic liver failure**

*Definition: The presence of one or more of the following:*

- *portal hypertension*
- *cirrhosis proved by biopsy, CT scan or ultrasound*
- *history of variceal bleeding*
- *hepatic encephalopathy in the past medical history*
- Ascites (non-malignant) in past medical history

4. **History of chronic renal failure**

*Definition: Need for chronic renal support or S-creatinine > 3.6 g/dL / 300 μmol/L prior to hospital admission.*

5. **Diabetes**

*Definition: Treatment at the time of hospital admission with any relevant drug indicating diabetes, e.g., insulin, alpha-glucosidase inhibitors, biguanides (metformin), dipeptidyl peptidase-4 inhibitors (e.g., Januvia), Glucagon-like peptide-1 receptor agonists, meglitinides, sodium-glucose transporter 2 inhibitors, sulfonylureas, thiazolidinediones.*

**Treatments before ICU admission**

- Treatment with a prokinetic agent

*Definition: Treatment with a prokinetic agent (erythromycin, metoclopramide, domperidone or prucolapride)*

- Treatment with parenteral nutrition

*Definition: Treatment with parenteral nutrition on the ICU Day of inclusion (within 24 hours of ICU admission).*

**Simplified Mortality Score for the Intensive Care Unit (SMS-ICU) (not included in the baseline variables)^1^**

1. **Lowest systolic blood pressure (mmHg)**

*Worst value registered in the 24 h prior to inclusion*

2. **Hematologic malignancy or metastatic cancer**

*History of malignant hematologic disorder or metastatic cancer in medical records.*

3. **Vasopressor/inotropes**

*Continuous use of vasopressors/inotropes (i.e., not boluses) on the ICU day of inclusion*

*(E.g., epinephrine, norepinephrine, dobutamine, dopamine, phenylephrine, metaraminol, milrinone, levosimendan, angiotensin II, and vasopressin or one of its analogues)*

4. **Respiratory support**

*Use of respiratory support, including invasive or non-invasive respiratory support and continuous use of continuous positive airway pressure (CPAP) on the ICU day of inclusion.*

5. **Renal replacement therapy**

*Use of any renal replacement therapy, whether chronic or acute, including continuous renal replacement therapy and intermittent haemodialysis, including up to three days between intermittent haemodialysis on the ICU day of inclusion.*

^1^ *Variables for the SMS-ICU score not otherwise included in the baseline variables*

*ICU: Intensive Care Unit, GI: gastrointestinal, NYHA: New York Heart association, LVEF: left ventricular ejection fraction, CT: computed tomography scan*

**Potential Serious adverse events**

- **Cardiac arrest**
- **Cardiac arrhythmias requiring pharmacological treatment**

*Definition: Any cardiac arrhythmia requiring pharmacological treatment, including supraventricular tachycardia, ventricular tachycardia, ventricular fibrillation, or bradycardia*

- **Extrapyramidal symptoms requiring pharmacological treatment**

*Definition:* *Akathisia, dystonia, parkinsonism, or tardive dyskinesia* *requiring pharmacological treatment*

- **Severe diarrhoea requiring treatment**

*Definition: A volume of ≥ 1000 ml/day and/or if severe diarrhoea is noted in the medical record and requires treatment, e.g., fluids, stopping/pausing medication, or pharmacological therapy.*

- **Vomiting with clinically significant aspiration requiring treatment**

*Definition: Entry of solid material into the trachea and lungs, resulting in clinical deterioration with need for treatment (pharmacological or non-pharmacological) and/or aspiration requiring treatment, was noted in the medical record.*

### S3 Simplified Mortality Score for the Intensive Care Unit (SMS-ICU)

|  | **Points** |
| --- | --- |
| **Age** | |
| ≤ 39 years  40 – 59 years  60 – 79 years  ≥ 80 years | 0  5  10  13 |
| **Lowest systolic blood pressure** | |
| ≤ 49 mmHg  50 – 69 mmHg  70 – 89 mmHg  ≥ 90 mmHg | 6  5  3  0 |
| **Acute surgical admission** | |
| No  Yes | 3  0 |
| **Haematological malignancy or metastatic cancer** | |
| No  Yes | 0  7 |
| **Vasopressors/inotropes^1^** | |
| No  Yes | 0  4 |
| **Respiratory support^2^** | |
| No  Yes | 0  5 |
| **Renal replacement therapy^3^** | |
| No  Yes | 0  4 |
| **Total score** | 0-42^4^ |

| 0  3  4  5  6  7  8  9  10  11  12  13  14  15  16  17  18  19  20  21 | 3.3%  4.8%  5.5%  6.2%  7.1%  8.0%  9.1%  10.3%  11.6%  13.1%  14.7%  16.5%  18.4%  20.5%  22.8%  25.3%  28.0%  30.8%  33.8%  36.9% |
| --- | --- |
| 22  23  24  25  26  27  28  29  30  31  32  33  34  35  36  37  38  39  41  42 | 40.1%  43.4%  46.7%  50.1%  53.5%  56.9%  60.2%  63.4%  66.4%  69.4%  72.2%  74.8%  77.3%  79.6%  81.7%  83.7%  85.4%  87.0%  89.8%  91.0% |

**Total score and predicted 90-day mortality risk**

*Reproduced from a previous paper.* (1)

*^1^ Continuous use of any vasopressor or inotrope.*

*^2^ Use of respiratory support, including invasive or non-invasive respiratory support and continuous use of continuous positive airway pressure (CPAP). Intermittent use of CPAP is not considered respiratory support.*

*^3^Use of renal replacement therapy includes any renal replacement therapy, whether chronic or acute, including continuous renal replacement therapy and intermittent haemodialysis, including the days in between intermittent haemodialysis.*

*^4^ Points assigned for the different variables in the score. It is not possible to obtain a total score of 1, 2 or 40 points. The worst value recorded during the first day in the ICU is used.* (1,2)

### S4 Baseline table

**Table S1 Baseline characteristics**

| **Characteristics** | **No SAEs**  N = 1,086 | **One or more SAEs** N = 354 |
| --- | --- | --- |
| Age (years) | 63 (47-74) | 68 (57-76) |
| Sex (male) | 619 (57.0%) | 200 (56.5%) |
| **Comorbidities** |  |  |
| Pulmonary disease | 192 (17.7%) | 83 (23.4%) |
| IHD or HF | 174 (16.0%) | 93 (26.3%) |
| Chronic renal failure | 91 (8.4%) | 44 (12.4%) |
| Chronic liver failure | 52 (4.8%) | 16 (4.5%) |
| Diabetes | 285 (26.2%) | 104 (29.4%) |
| Haematologic malignancy or metastatic cancer | 80 (7.4%) | 47 (13.3%) |
| **Source of admission** |  |  |
| Emergency department | 548 (50.5%) | 138 (39.0%) |
| General ward | 277 (25.5%) | 126 (35.6%) |
| Operating/recovery room | 200 (18.4%) | 69 (19.5%) |
| Another ICU | 61 (5.6%) | 21 (5.9%) |
| **Surgery** |  |  |
| Elective surgery | 132 (12.2%) | 42 (11.8%) |
| Acute surgery  Abdominal surgery  Bariatric surgery | 263 (24.2%)  129 (11.9%)  10 (0.9%) | 93 (26.3%)  59 (16.7%)  4 (1.1%) |
| **Illness severity** |  |  |
| SMS-ICU^a^  Predicted 90%-day mortality (%)^b^ | 16 (12-21)  22.8 (14.7-36.9) | 22 (17-25)  40.1 (25.3-50.1) |
| **Main reason for ICU admission** |  |  |
| Neurological condition | 245 (22.6%) | 41 (11.6%) |
| Respiratory failure | 276 (25.4%) | 83 (23.4%) |
| Circulatory failure | 225 (20.7%) | 151 (42.7%) |
| Renal failure | 38 (3.5%) | 13 (3.7%) |
| Liver failure | 21 (1.9%) | 2 (0.6%) |
| Gastrointestinal bleeding | 28 (2.6%) | 10 (2.8%) |
| Other | 253 (23.3%) | 54 (15.3%) |
| **Prior treatment** |  |  |
| Prokinetic use prior to ICU admission | 47 (4.3%) | 15 (4.2%) |
| Parenteral nutrition prior to ICU admission | 39 (3.6%) | 23 (6.5%) |
| **Country** |  |  |
| Denmark | 484 (44.6%) | 157 (44.4%) |
| Saudi Arabia | 145 (13.4%) | 48 (13.6%) |
| Sweden | 98 (9.0%) | 16 (4.5%) |
| Finland | 77 (7.1%) | 18 (5.1%) |
| Poland | 49 (4.5%) | 41 (11.6%) |
| United Kingdom | 56 (5.2%) | 19 (5.4%) |
| Spain | 56 (5.2%) | 14 (4.0%) |
| Estonia | 44 (4.1%) | 19 (5.4%) |
| Netherlands | 43 (4.0%) | 12 (3.4%) |
| Kuwait | 24 (2.2%) | 6 (1.7%) |
| Switzerland | 10 (0.9%) | 4 (1.1%) |

***Table S1*** *Missing baseline data (7 patients, 0.5% in total): blood pressure: two patients (0.1%); information on elective surgery: one patient (0.1%); information on one comorbidity: three patients (0.2 %); information on parenteral nutrition: one patient (0.1%) (detailed in supplementary material 7).*

*Continuous variables are presented as medians with interquartile range (IQR) and categorical variables as counts and percentages. Definitions of baseline variables are available in the supplementary material 2.*

*^a^ Median raw scores (IQR).*

*^b^ Predicted 90-day mortality based on the SMS-ICU (an illness severity score ranging from 0-42, details in supplementary material 3).*

*IHD: Ischemic Heart Disease, HF: Heart Failure, OR: Operating or Recovery Room, ICU: Intensive Care Unit, SMS-ICU: Simplified Mortality Score for the Intensive Care Unit*

### S5 Overview of predefined SAEs

**Table S2 SAEs in the cohort**

| Type of SAE | Number of events | Number of patients |
| --- | --- | --- |
| Cardiac arrhythmias | 340 | 199 |
| Cardiac arrest | 173 | 158 |
| Diarrhoea | 119 | 56 |
| Vomiting with aspiration | 63 | 45 |
| Extrapyramidal symptoms | 15 | 13 |

**Table S2.** *Overview of SAEs in the cohort regardless of prokinetic treatment. In the number of events, the same patient may appear multiple times if they have experienced more than one SAE.*

### S6 SAEs and treatment duration

**Table S3 Number of patients experiencing each predefined SAE after prokinetic treatment, stratified by treatment duration**

| SAE type | ≤3 days; n= number of patients (% of total 128 patients) | >3 days; n=number of patients  (% of total 59 patients) |
| --- | --- | --- |
| Cardiac arrest | 11 (8.5%) | 7 (11.9%) |
| Cardiac arrhythmias | 12 (9.3%) | 6 (10.2%) |
| Diarrhoea | 7 (5.4%) | 5 (8.5%) |
| Vomiting | 4 (3.1%) | 1 (1.7%) |
| Extrapyramidal symptoms | 2 (1.6%) | 1 (1.7%) |

**Table S3.** *Numbers represent the number of patients with each SAE, stratified by treatment duration. Percentages are calculated relative to the total number of patients in each duration group (≤3 days: n = 138; >3 days: n = 49). A patient may contribute to multiple SAE types but is counted only once per type. For the ≤3-day group, SAEs were counted only if they occurred after treatment discontinuation; for the >3-day group, SAEs were counted from treatment day 4 onward*

### S7 Missing data

**Table S4 Missing baseline variables**

| Variable | Missing n (%) |
| --- | --- |
| Blood pressure | 2 (0.1%) |
| Chronic liver failure | 1 (0.1%) |
| Chronic pulmonary disease | 1 (0.1%) |
| Ischemic heart disease or heart failure | 1 (0.1%) |
| Parenteral nutrition at ICU admission | 1 (0.1%) |
| Elective surgery | 1 (0.1%) |

**Table S4.** *Seven patients had one missing baseline variable (0.5%). For those with missing blood pressure, no measurement was recorded within the first 24 hours of ICU admission. Only variables with missing values are included in the table.*

*ICU: Intensive Care Unit*

**Table S5 Missing daily variables**

| Daily variable | Number of patients | Number of ICU days with missing data |
| --- | --- | --- |
| Infusion of vasopressor | 1 | 1 |
| Invasive mechanical ventilation | 1 | 1 |
| Renal replacement therapy | 1 | 1 |
| Cardiac arrhythmias | 1 | 1 |
| Extrapyramidal symptoms | 1 | 1 |

**Table S5.** *Three patients (0.2%) had one or more daily variables missing. In one patient, all life support variables were missing for a single intensive care unit day. Only variables with missing values are shown.*

*ICU: Intensive Care Unit*

### References

1.	Granholm A, Perner A, Krag M, Hjortrup PB, Haase N, Holst LB, et al. Development and internal validation of the Simplified Mortality Score for the Intensive Care Unit (SMS-ICU). Acta Anaesthesiologica Scandinavica [Internet]. 2018 [cited 2022 Dec 6]; Available from: https://onlinelibrary.wiley.com/doi/10.1111/aas.13048

2.	Granholm A, Perner A, Krag M, Marker S, Hjortrup PB, Haase N, et al. External validation of the Simplified Mortality Score for the Intensive Care Unit (SMS-ICU). Acta Anaesthesiologica Scandinavica. 2019;63(9):1216–24.
